# Construction and validation of a database of head models for functional imaging of the neonatal brain

**DOI:** 10.1002/hbm.25242

**Published:** 2020-10-17

**Authors:** Liam H. Collins‐Jones, Tomoki Arichi, Tanya Poppe, Addison Billing, Jiaxin Xiao, Lorenzo Fabrizi, Sabrina Brigadoi, Jeremy C. Hebden, Clare E. Elwell, Robert J. Cooper

**Affiliations:** ^1^ DOT‐HUB, Department of Medical Physics and Biomedical Engineering University College London London UK; ^2^ Biomedical Optics Research Laboratory, Medical Physics and Biomedical Engineering University College London London UK; ^3^ Centre for the Developing Brain, Division of Imaging Sciences and Biomedical Engineering King's College London, King's Health Partners, St Thomas' Hospital London UK; ^4^ Department of Bioengineering Imperial College of Science, Technology, and Medicine London UK; ^5^ Institute for Cognitive Neuroscience University College London London UK; ^6^ Department of Neuroscience, Physiology and Pharmacology University College London London UK; ^7^ Department of Information Engineering University of Padova Padova Italy; ^8^ Department of Developmental Psychology and Socialisation University of Padova Padova Italy

**Keywords:** atlas, database, neonatal, structural prior

## Abstract

The neonatal brain undergoes dramatic structural and functional changes over the last trimester of gestation. The accuracy of source localisation of brain activity recorded from the scalp therefore relies on accurate age‐specific head models. Although an age‐appropriate population‐level atlas could be used, detail is lost in the construction of such atlases, in particular with regard to the smoothing of the cortical surface, and so such a model is not representative of anatomy at an individual level. In this work, we describe the construction of a database of individual structural priors of the neonatal head using 215 individual‐level datasets at ages 29–44 weeks postmenstrual age from the Developing Human Connectome Project. We have validated a method to segment the extra‐cerebral tissue against manual segmentation. We have also conducted a leave‐one‐out analysis to quantify the expected spatial error incurred with regard to localising functional activation when using a best‐matching individual from the database in place of a subject‐specific model; the median error was calculated to be 8.3 mm (median absolute deviation 3.8 mm). The database can be applied for any functional neuroimaging modality which requires structural data whereby the physical parameters associated with that modality vary with tissue type and is freely available at www.ucl.ac.uk/dot-hub.

## Graphical abstract

When no subject‐specific structural data are available, the accuracy of source localisation of brain activity recorded from the scalp relies on accurate age‐specific head models. In this work, we describe the construction of a database of individual structural priors of the neonatal head using 215 individual‐level datasets at ages 29–44 weeks postmenstrual age from the Developing Human Connectome Project. We also present the results of a leave‐one‐out analysis to validate the accuracy of using a matched model from the database in place of a subject‐specific model.

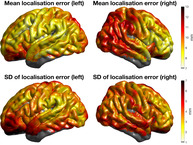

## 1 INTRODUCTION

A structural prior is a model of structural anatomy that delineates the different tissues within a particular anatomical region. Structural priors of the cranial anatomy can be beneficial to a wide range of neuroimaging and neuromodulation modalities that monitor brain function via neuronally generated electromagnetic fields (electronencephalography [EEG]; and magnetoencephalography [MEG]), or via haemodynamics (such as functional near‐infrared spectroscopy [fNIRS], diffuse correlation spectroscopy, photoacoustic imaging) but do not simultaneously acquire structural data.

Functional magnetic resonance imaging (fMRI) has been used extensively to study functional development of the neonatal brain, for example, by mapping the sensorimotor cortex (Allievi et al., 2016; Arichi et al., 2010; Arichi et al., 2012; Dall'Orso et al., 2018). However, given the requirement that subjects remain still, fMRI cannot be used to study the awake infant, which limits fMRI data acquisition to infants who are either asleep or sedated.

Such constraints can be overcome by using other more motion tolerant functional imaging techniques to collect functional data, such as fNIRS, an optical imaging technique whereby the head is interrogated with near‐infrared light via an array of sources and detectors placed on the scalp (Lee, Cooper, & Austin, 2017; White, 2010). Changes in the detected light intensity measured between a resting state and a stimulated state are used to calculate changes in oxy‐ and deoxy‐haemoglobin concentration in the cortex, which are markers of functional activation. Advantages of fNIRS include the fact that it is silent, non‐invasive, portable, and relatively tolerant of motion (Eggebrecht et al., 2014; Ferradal et al., 2016; Lee et al., 2017). However, techniques such as fNIRS conventionally offer limited spatial resolution (Lloyd‐Fox, Blasi, & Elwell, 2010), but can be extended to produce three‐dimensional images with the use of a structural prior.

Structural priors are often derived from magnetic resonance imaging (MRI) data, given its high soft tissue contrast and high spatial resolution (Makropoulos, Counsell, & Rueckert, 2018; Makropoulos, Robinson, et al., 2018). The tissues that must be delineated will depend on the application modality, how the physical parameters associated with that modality vary with tissue type, and how well known those parameters are. Common segmentations include grey matter, white matter, and cerebrospinal fluid (CSF). However, since the brain does not exist in isolation, fields induced or detected on the scalp surface must propagate through non‐brain tissues to reach the brain. These tissues, predominantly the skull and scalp, must also be represented in a structural prior. Structural priors can be used to support image registration (Xiao et al., 2016), improve the targeting of neuromodulation (Mueller, Ai, Bansal, & Legon, 2017), or to model the spatial distribution of the electric, magnetic, ultrasonic or optical fields that underpin many imaging modalities (Arridge & Cooper, 2015; Azizollahi, Aarabi, & Wallois, 2016; Legon et al., 2018; Pirondini et al., 2018; Ranjbaran et al., 2019; Roche‐Labarbe et al., 2008; Routier et al., 2017). As such, structural priors are needed to improve the spatial specificity of functional imaging modalities.

One modality where structural priors of the head are particularly important is diffuse optical tomography (DOT), an extension of fNIRS in which optical data is used in conjunction with a structural prior to reconstruct three‐dimensional images of haemoglobin concentration changes in the cortex. By using a structural prior to model field propagation within the head, imaging techniques such as DOT are enabled which produce images that spatially localise activation on the cortex (rather than relying on the position of sources and detectors on the scalp to interpret results, as is done in conventional fNIRS). In the last two decades, a large body of research applying DOT to the study of the neonatal brain has been established, such as studies of functional activation (Austin et al., 2006; Hebden, 2003; Karen et al., 2019; White, Liao, Ferradal, Inder, & Culver, 2012), neuropathology (Chalia et al., 2016; Dempsey et al., 2014; Plomgaard et al., 2016; Singh et al., 2016), and monitoring the brain for extended periods (Brigadoi et al., 2019). Previous work with infants has also employed prior structural information as a space to which functional data can be registered (Papademetriou et al., 2013).

As DOT measurements provide no structural information about the target object, an appropriate structural prior is critical. The more realistic the structural priors, the higher the accuracy of the photon propagation model that underpins image reconstruction, and therefore the more accurate the reconstructed images (Ferradal, Eggebrecht, Hassanpour, Snyder, & Culver, 2014). To achieve the highest accuracy, an individual's own MR image can be used to produce a subject‐specific structural prior (Cooper et al., 2012). Despite offering the highest accuracy, necessitating an MRI scan for every subject undermines many of the advantages of DOT, such as its portability and tolerance of motion. What can be used in place of a subject‐specific model is an atlas: a structural prior based on MRI data acquired from other individuals. Atlases can consist of spatially averaged MRI data from a group of individuals from a population of interest, which are intended to be representative of that population (Brett, Johnsrude, & Owen, 2002; Tsuzuki & Dan, 2014). We will refer to such atlases here as *population‐level* atlases. In contrast, an individual atlas is a structural prior that derives from the MRI data of a single individual, be that from just one scan or (as is the case for the Colin27 atlas (Collins et al., 1998)) by spatially averaging several scans from the same individual. In order for an atlas to be applied, it will usually first be spatially registered to a given subject using cranial landmarks, which can be measured on the subject using a digitising positioning system or via photogrammetric methods (Tsuzuki & Dan, 2014). Several MRI atlases have been (and continue to be) applied for use in DOT of adults. These models include the adult MNI152 population‐level atlas (Custo et al., 2010) and the Colin27 individual atlas (Aasted et al., 2015; Cooper et al., 2012).

The use of structural priors for infants and children has proved much more challenging to implement. Due to the rapid maturation of the neonatal brain (Makropoulos et al., 2016), selecting structural priors matched by age is critical. Atlases derived from adult data do not accurately represent the patterns of maturation seen in the developing brain, and so the infant brain cannot simply be treated as a smaller version of the adult (Fonov et al., 2011; Richards, Sanchez, Phillips‐Meek, & Xie, 2016; Richards & Xie, 2015). However, historically there has been a lack of publicly available structural neonatal MRI data (Makropoulos, Counsell, & Rueckert, 2018) which has limited the production of infant brain models.

Despite these challenges, several MRI brain atlases have been produced for neonates in the last decade. Heiskala, Pollari, Metsäranta, Grant, and Nissilä (2009), Oishi et al. (2011), and Shi et al. (2011) have all proposed spatial averaging of MRI data to produce a population‐level atlas for neonates, however the resulting atlases do not encode any measure of postmenstrual age (PMA). However, given that the neonatal brain is rapidly developing, a more appropriate approach is to produce population‐level atlases for specific age points throughout the neonatal period. This was the method adopted by Kuklisova‐Murgasova et al. (2011) who produced age‐specific population‐level MRI templates and tissue probability maps for infants aged 29–44 weeks PMA at 1‐week intervals. Serag et al. (2012) also produced an age‐dependent atlas for a similar age range—28–44 weeks PMA—using data from 204 neonatal infants non‐rigidly registered to age‐specific common spaces. Makropoulos et al. (2016) took a similar approach to produce an average atlas using data from 420 images taken from infants aged 27–45 weeks PMA, and also include a parcellation atlas delineating 82 brain structures.

A major barrier to these infant atlases being used as spatial priors for portable imaging modalities such as DOT is that they do not routinely contain skull and scalp tissues. A major application of these MRI atlases is usually to aid automated brain tissue segmentation procedures (Makropoulos, Counsell, & Rueckert, 2018) and, as a result, the skull and scalp tissues need not to be considered. To overcome this issue, Brigadoi, Aljabar, Kuklisova‐Murgasova, Arridge, and Cooper (2014) produced a population‐level atlas of four‐layer tissue models, consisting of grey matter; white matter; CSF and extra‐cerebral tissue (a combined label for skull and scalp) for infants aged 28–44 weeks PMA at 1‐week intervals. This atlas was constructed using age‐specific tissue probability maps from the Kuklisova–Murgasova atlas to produce a three‐layer model for brain tissues. Skull and scalp tissues are difficult to separate on neonatal MRI due to their low thickness and lack of differential contrast (Brigadoi et al., 2014). However, because these tissues have relatively similar optical properties (Dehaes et al., 2013), Brigadoi et al. produced a combined segmentation for these two tissues (referred to as extra‐cerebral tissue, using the Betsurf procedure (Jenkinson, Pechaud, & Smith, 2005). This atlas was the first of its kind, and has been freely disseminated to researchers in a wide range of fields. It has been applied in neonatal atlas‐guided DOT (Chalia et al., 2016; de Oliveira et al., 2019; Singh et al., 2014; Verriotis et al., 2016), and photoacoustic modelling (Ranjbaran et al., 2019), and optical phantom construction (Dempsey, Persad, Powell, Chitnis, & Hebden, 2017).

However, a major limitation of the Brigadoi et al. head model is that neither the accuracy of the extra‐cerebral tissue segmentation, nor the spatial error in activation localisation incurred by the use of their model in place of a subject‐specific prior, could be directly assessed. This was because the associated individual‐level MRI data upon which their model was based was not publicly available. Nor was there an existing *gold‐standard* model against which to compare. The authors noted that it is likely that their atlas underestimates the thickness of the extra‐cerebral tissue. A further issue that is true of the Brigadoi model, and of many population‐level atlases, is that detail is lost in the process of spatially averaging the MRI data (Makropoulos, Counsell, & Rueckert, 2018). Given its highly variable folding pattern, the cortical surface tends to become smoothed following spatial averaging, and a similar smoothing effect is seen on the detail of the white matter. The resulting spatially averaged models are therefore not representative of any single individual, and may therefore not offer an anatomically meaningful space in which the spatial distribution of a physical field can be modelled. In addition, surface registration techniques such as the well‐established FreeSurfer software (Fischl, Sereno, & Dale, 1999) are increasingly used to permit comparison between subjects and groups in functional neuroimaging and have been incorporated as part of the structural pipeline of the Human Connectome Project (Glasser et al., 2013). However, the use of atlases with smoothed cortical surfaces is often incompatible with surface‐based registration techniques, which can limit the development of new data analysis pipelines.

The Developing Human Connectome Project (dHCP) is acquiring structural, functional and diffusion MRI data from neonatal infants and foetuses to build the first spatio‐temporal connectome of early life (www.developingconnectome.org). MRI data from the dHCP has recently been released, including structural images and brain tissue segmentations. In this work, we take advantage of this newly available structural data from the dHCP to produce a database of multi‐layered structural priors of the neonatal head, including an extra‐cerebral tissue layer, for individuals aged 29–44 weeks PMA for use in DOT and potentially several other imaging modalities. We define an extra‐cerebral tissue segmentation method and then validate that approach across our database, and describe a package of multi‐layer tissue masks and meshes. Finally, using a leave‐one‐out analysis, we quantify the spatial error incurred by one possible application of this database: using an age and size‐matched individual in place of subject‐specific structural model.

## 2 METHODS

### 2.1 Model construction

#### 2.1.1 Structural MRI data

The MR images were acquired as part of the dHCP, whose resources have been made open‐source (www.developingconnectome.org). Images were obtained from infants using a 3 T Philips Achieva Scanner (Best, NL) at the Evelina Newborn Imaging Centre, St Thomas' Hospital, London, with a 32‐channel dedicated neonatal head coil (Hughes et al., 2017). The T2‐weighted images were acquired in two stacks of slices acquired in sagittal and axial planes using parameters TR = 12 s, TE = 156 ms, SENSE factor 2.11 (axial), and 2.58 (sagittal) (Makropoulos, Robinson, et al., 2018). Overlapping slices (resolution 0.8 × 0.8 × 1.6 mm^3^) were acquired, and the final up‐sampled image resolution was 0.5 × 0.5 × 0.5 mm^3^ after reconstruction and motion correction (Cordero‐Grande, Hughes, Hutter, Price, & Hajnal, 2018; Cordero‐Grande et al., 2016; Kuklisova‐Murgasova et al., 2011). All T1‐weighted images were acquired using an inversion recovery sequence at the same resolutions using parameters TI = 1740 ms, TR = 4.8 s, TE = 8.7 ms, SENSE factor 2.26 (axial) and 2.66 (sagittal). All images were reviewed by a paediatric neuroradiologist.

Datasets from 634 individuals whose images had undergone the dHCP structural processing pipeline (Makropoulos et al., 2014; Makropoulos, Robinson, et al., 2018) were used to construct structural priors in this work. The datasets included binary tissue segmentations for brain tissues (cortical grey matter, white matter, outer CSF, ventricles, deep grey matter, hippocampus, brainstem, and cerebellum) with voxel dimensions: 0.5 × 0.5 × 0.5 mm^3^, as well as T1‐ and T2‐weighted MR images (voxel dimensions: 0.5 × 0.5 × 0.5 mm^3^) for each individual. An evaluation of the quality of the brain tissue segmentations can be found in Makropoulos, Robinson, et al. (2018).

**FIGURE 1:**
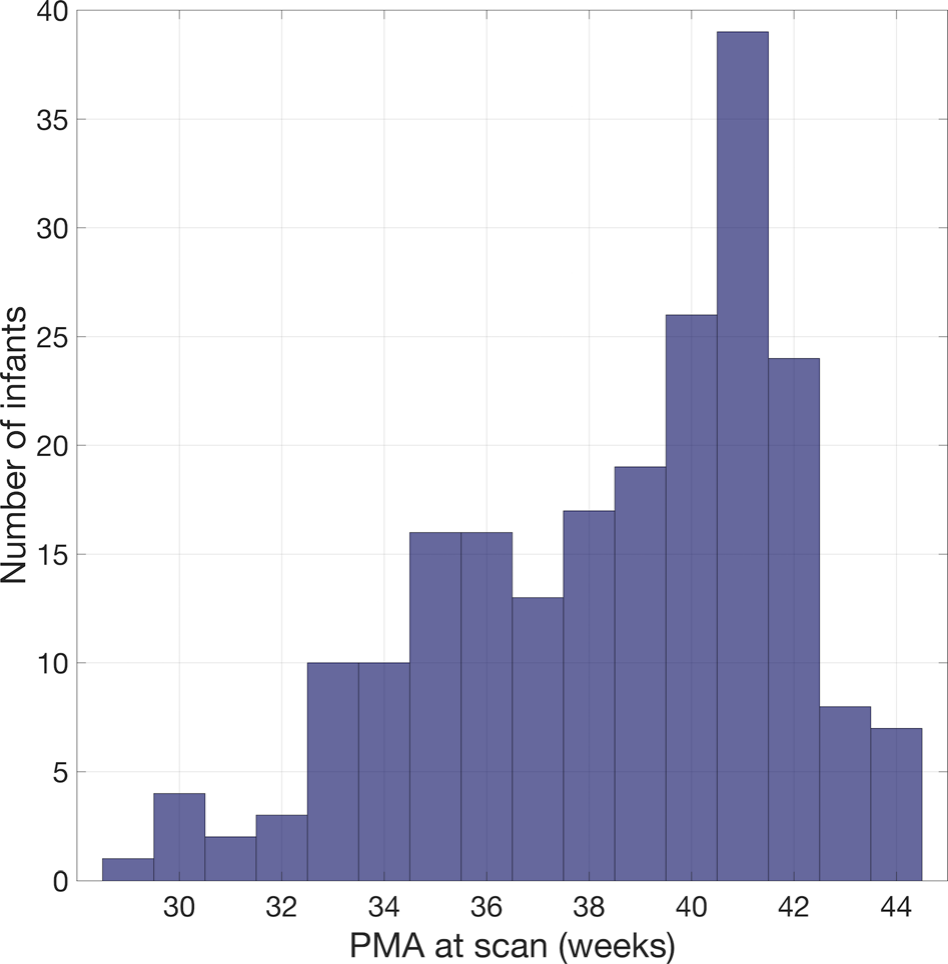
Age distribution of infants whose structural data were used to produce the database of neonatal structural priors

Each individual's T1‐weighted volume was inspected visually to determine whether the dataset would be appropriate for producing a multi‐layer tissue model. Datasets were excluded if extra‐cerebral tissue was cropped from the image superiorly or laterally (this excluded 314 subjects); the pre‐auricular points were not in the field of view of the T1‐weighted image (excluded 15 subjects); severe motion artefact was present in the image that significantly distorted the outer scalp boundary (excluded 24); or a haematoma was present in the extra‐cerebral tissue that impacted the outline of the outer scalp boundary (excluded 13). This process identified a total of 268 datasets deemed appropriate for inclusion. However, at present only 215 of these datasets have been made publicly available by the dHCP and so only structural data deriving from these publicly available datasets can be included in our database. Figure 1 shows the age distribution of these 215 individuals.

#### 2.1.2 Segmentation of the extra‐cerebral tissue

No *gold standard* exists for the segmentation of skull and scalp layers for the neonatal MRI. Though studies have been published that propose methods to segment these tissues (Daliri et al., 2010; Ghadimi et al., 2008), these studies relied on manual segmentation of a small group of individuals. As a result, in this work, a similar strategy to Brigadoi et al. was pursued and so a segmentation approach for the scalp and skull in combination (i.e., the extra‐cerebral tissue) was developed.

For each of the selected datasets, the pre‐defined segmentations for brain tissues and CSF were combined and thresholded to produce a binary cerebral tissue mask, the outer extent of which was used to define the inner skull boundary. In order to define an extra‐cerebral tissue mask, a method was needed to demarcate the outer scalp boundary. Once determined, the shape defined by this boundary could be filled in the axial, sagittal and coronal planes to produce a head tissue mask. The cerebral tissue mask is then subtracted from the head tissue mask to produce the extra‐cerebral tissue mask.

In this work, we investigated different approaches to segment extra‐cerebral tissue, which in our case depended on determining the outer scalp boundary. One method investigated was Betsurf: a well‐established tool that uses the intensity distribution of an MR image constrained to a robust range, to find the scalp surface (Jenkinson et al., 2005). Betsurf can be run with an individual's T1‐weighted image as its only input, or it can use both a T1‐ and T2‐weighted image from the same individual. In this work, both of these implementations of Betsurf were evaluated. Another method investigated was Otsu thresholding (Otsu, 1979), a method that fits a bimodal model to the distribution of image intensities to determine a threshold between foreground and background voxels. Otsu thresholding has been previously employed by (Tuan, Kim, & Bao, 2018) to define the outer scalp boundary in an adult model. Using each of these methods, an outer scalp boundary was delineated and a one‐voxel thick boundary was then extracted.

In order to quantify performance and determine the appropriateness of these different methods, a validation approach involving manual segmentation was employed. The air‐tissue boundary for a subset of 12 individuals' MRIs were manually segmented. The subset of 12 consisted of 3 arbitrarily chosen infants at four age‐points (32, 36, 40, and 44 weeks PMA). For the manual segmentation itself, T1‐weighted images were used, which offered better contrast for scalp tissue than T2‐weighted images. Manual segmentations were completed slice‐by‐slice in the axial plane then reviewed and modified in the sagittal and coronal planes. The segmentations were then filled slice‐by‐slice in the axial plane, before extracting a one‐voxel thick outer boundary. All 12 manual segmentations were completed by a single rater (and validated by two independent raters, see below), using ITK‐SNAP (Yushkevich & Gerig, 2017). Infants were selected from a range of ages spanning 12 weeks PMA to identify if there was any relationship between segmentation method performance and age, which could lead us to determine whether a single segmentation method was appropriate at all ages or whether different segmentation methods were needed to be applied at different ages.

For each individual, the one‐voxel thick outer scalp boundaries from the manual segmentation were compared to each of the segmentations resulting from the automated methods. Three different metrics were used to compare each manual and automated segmentation. The mean surface distance (the mean of the distances from/to the centre of each manually segmented voxel to/from the centre of the nearest automated segmentation voxel) was used to provide an overall measure of similarity. The Hausdorff distance (the maximum of these distances) was used to quantify the maximum error, while the modified Hausdorff distance (the value of the 95th percentile of these distances) was used to quantify the spread of error in the data without being biased towards outliers.

In order to obtain a measure of inter‐rater variability, 2 of the 12 images were manually segmented by two additional independent raters, and the similarity with the primary rater was compared using the metrics outlined above. The mean surface distance between the primary rater's segmentation and those completed by the other two raters was 0.153 mm with a *SD* of 0.236 mm. The Hausdorff distance was 1.5 mm and the modified Hausdorff distance was 0.5 mm.

#### 2.1.3 Creating a multi‐layer tissue mask

**FIGURE 2:**
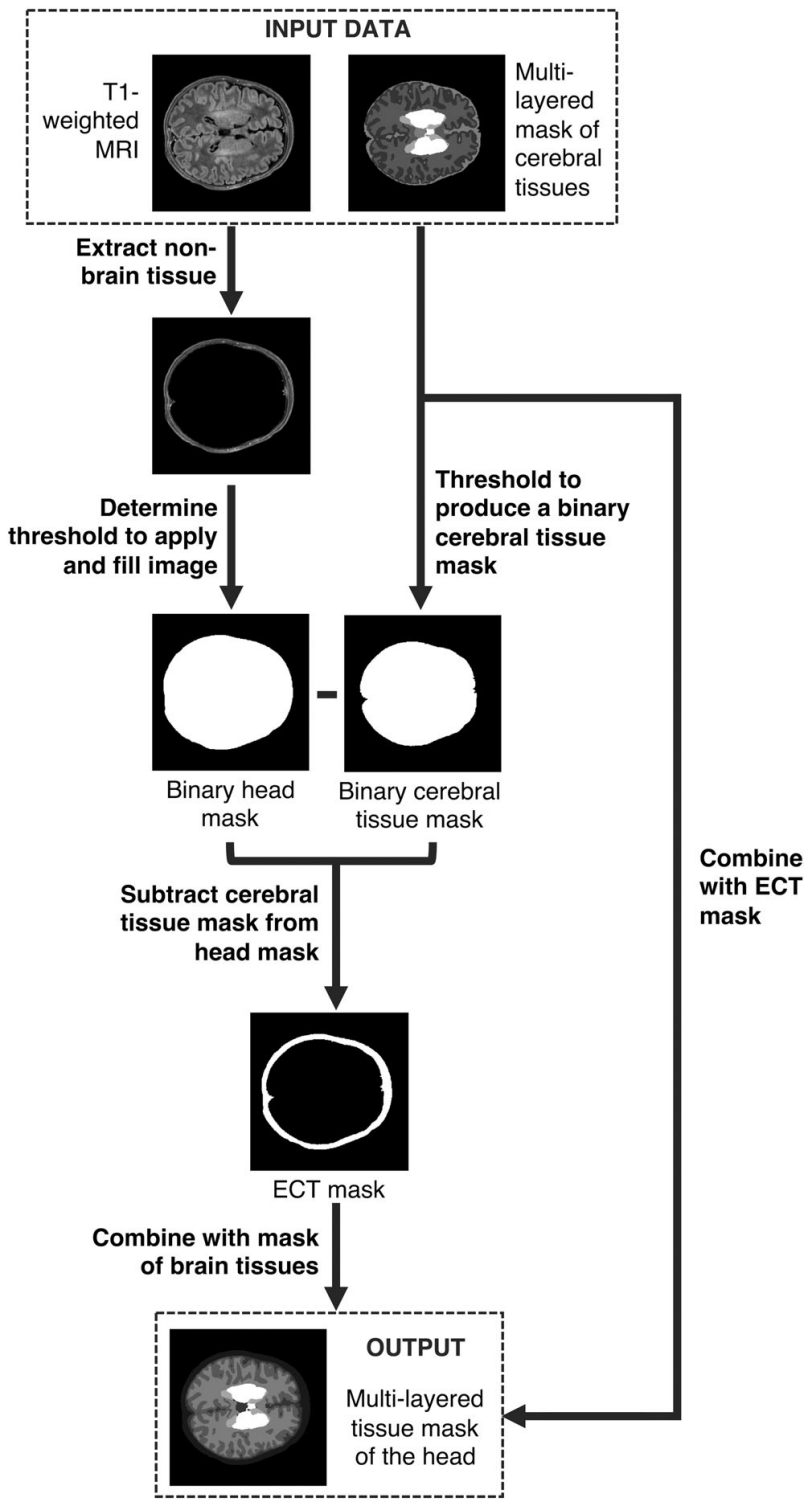
Flowchart describing the construction of structural priors for each neonatal individual using segmentations of cerebral tissues and an extra‐cerebral tissue (ECT) segmentation extracted from a T1‐weighted MR image

Having established an appropriate automated procedure for the identification of the outer scalp boundary (see Section 3), the extra‐cerebral tissue segmentation could be combined with the cerebral tissue mask. The full segmentation pipeline, used to produce multi‐layer tissue masks for each of the 215 individuals, is outlined in Figure 2. When considering the optical properties of cerebral tissues, cortical and deep grey matter can be grouped to become a single tissue label, grey matter; ventricles and outer CSF can be grouped to become a single label, CSF; and white matter, brainstem, and cerebellum (which largely consists of white matter) can be grouped into one label, white matter.

#### 2.1.4 Constructing a volumetric tetrahedral mesh

A high‐density tetrahedral volumetric mesh was produced for each of the 215 datasets using the iso2mesh toolbox (Fang & Boas, 2009). The multi‐layered tissue masks were used as an input for a meshing procedure which assumes isotropic voxels: using the CGAL mesher option, the maximum element size was set to 1.5 unit cubed (where 1 unit is equal to 0.5 mm, the length of an isotropic voxel), while the maximum radius of the Delaunay sphere was set to 1 unit. The coordinates of the nodes of the resulting mesh were then rescaled into millimetre units. The quality of the volumetric meshes was assessed using the Joe–Liu quality index (Liu & Joe, 1994):

$$q_{\mathrm{vol}}=\frac{12∙{\left(3∙\mathrm{vol}\right)}^{\frac{2}{3}}}{\sum_{0\leq i\leq j\leq 3}l_{i,j}^{2}}$$

where *q*_*vol*_ is the Joe–Liu quality index, *vol* is the volume of a tetrahedron, and *l*_*i*,*j*_ are the lengths of the edges of the tetrahedron. Another metric, the mean Voronoi volume—the volume around a given mesh node which encompasses each point which is closer to the given node than any other mesh node—was used as an indication of mesh density; the lower the Voronoi volume, the higher the mesh density. In each tetrahedral volume mesh, the tissue labels (indexed from 1 to 9 specifying extra‐cerebral tissue, outer CSF, cortical grey matter, white matter, ventricles, cerebellum, deep grey matter, brainstem, and hippocampus, respectively) were computed on an element‐wise basis.

#### 2.1.5 Cranial landmarks and 10–5 positions

A convention for describing positions on the scalp surface of each model is very helpful in determining equivalent scalp surface positions across different subjects, for spatially registering structural priors and for determining appropriate positions of optodes, electrodes or any other equipment placed on the scalp surface. The 10–5 system is a convention for describing positions on the scalp, originally intended as a convention for high‐density electrode positioning in EEG (Oostenveld & Praamstra, 2001). The 10–5 system is computed using the coordinates of the cranial landmarks, corresponding to the nasion (Nz), the inion (Iz), the left pre‐auricular point (Al), the right pre‐auricular point (Ar), and the approximate location of the vertex of the head (Cz). For each individual in the database, these five landmarks were determined manually: ITK‐SNAP was used to provide a visualisation of the external surface of the extra‐cerebral tissue (i.e., the scalp) and a single voxel was selected to represent the location of each landmark. The coordinates of the node in the head volume mesh closest to the centre of that voxel were then taken as the updated coordinates of each landmark in the volume mesh space. Each node in the mesh was rigidly transformed to a coordinate system in which the origin is defined as Iz, the y‐axis is defined as a vector joining Iz to Nz, and mesh nodes are rotated around the y‐axis such that the z‐coordinates of Ar and Al are approximately equal.

For every individual, the coordinates of the 10–5 locations were then calculated using a curve‐walk procedure (Aasted et al., 2015) see Homer2: www.nitrc.org/projects/homer2). Given three points on the mesh surface, a plane can be computed. The coordinates for Nz, Iz, and Cz are used to define a plane and the intersection between the plane and the outer surface of the volumetric mesh (i.e., the scalp surface) is computed. As the intersection has been defined in the mesh space, a set of nodes at the scalp surface that lie within 0.6 mm of the intersection of the plane were identified to define a curve. A 3D spline interpolation of these surface nodes is then used to smooth the curve and avoid errors due to zigzagging between the nodes comprising the initial curve. The positions along the smoothed curve from Nz to Iz via Cz are then calculated by dividing the total length of the curve into 5% intervals. This curve is referred to as the sagittal reference curve. The same process as above is used to compute the coronal reference curve using points Ar, Al, and Cz to define the intersection with the scalp surface, and the positions along the curve are computed at 5% intervals. An axial reference curve is then defined for the entire circumference of the head by calculating two curves: using FPz, T7, and Oz on the left and FPz, T8, and Oz on the right, and dividing both curves into 5% intervals. The length of the axial reference curve is taken to be the head circumference. The remainder of the 10–5 positions are calculated along curves defined by equivalent positions on the right and left sides of the axial reference curve via equivalent positions in the sagittal reference curve.

To preserve anonymisation of the dHCP data, the tissues around the eyes in the MR images have been intentionally distorted. The scalp surface therefore could not be recovered for the anteroinferior‐most regions of the head. The 10–5 positions consist of 345 locations; however, the lower‐most curve of landmarks on the head (which lies below the axial reference curve) could not be reliably computed given the anterior distortion of the head. Due to this, all 10–5 positions below the axial reference curve were excluded. In addition, for each individual the manually determined cranial landmarks were saved but were not used in subsequent analyses in this study.

### 2.2 Performance of the model in an example application

While subject‐specific structural MRI data may not be available, knowledge of the subjects age and external features of the head may be available and are usually far easier to obtain. One such external feature could be head circumference. Other external features of the head could include the locations of landmarks on the scalp surface, for instance the cranial landmarks, derived using a three‐dimensional magnetic digital position tracking system (Tsuzuki & Dan, 2014) or by using photogrammetry methods (Lloyd‐Fox et al., 2014). This is often the case in both DOT and EEG studies of neonates. For a given subject, such characteristics can be used to choose a best‐matching individual from our neonatal head model database.

To demonstrate how this database can potentially be used, a simple example application and error quantification pipeline were developed. The functional imaging modalities outlined in this paper rely on the placement of equipment on the scalp surface. In our example application, scalp positions from a subject‐specific model and a matched model were projected to the cortical surface. These projection positions were compared to quantify the difference in cortical anatomy underlying equivalent scalp positions in the two models, which we use as a measure of the error incurred by using a matched model in place of a subject‐specific model.

For each individual, the 10–5 EEG positions were projected to the cortex. The nodes of the scalp surface of the volumetric mesh within a 5 mm radius of each 10–5 position were used to fit a plane. For a given 10–5 position, a ray vector was defined orthogonal to this plane. The ray was extended to find its intersection with a face on the cortical surface using the Möller–Trumbore algorithm (Mena‐Chalco, 2019; Möller & Trumbore, 1997). The projection position for 10–5 positions whose ray vector did not intersect the cortical surface were assigned to be the nearest cortical surface node to the 10–5 position. The hemisphere of the resulting projection was also noted.

**FIGURE 3:**
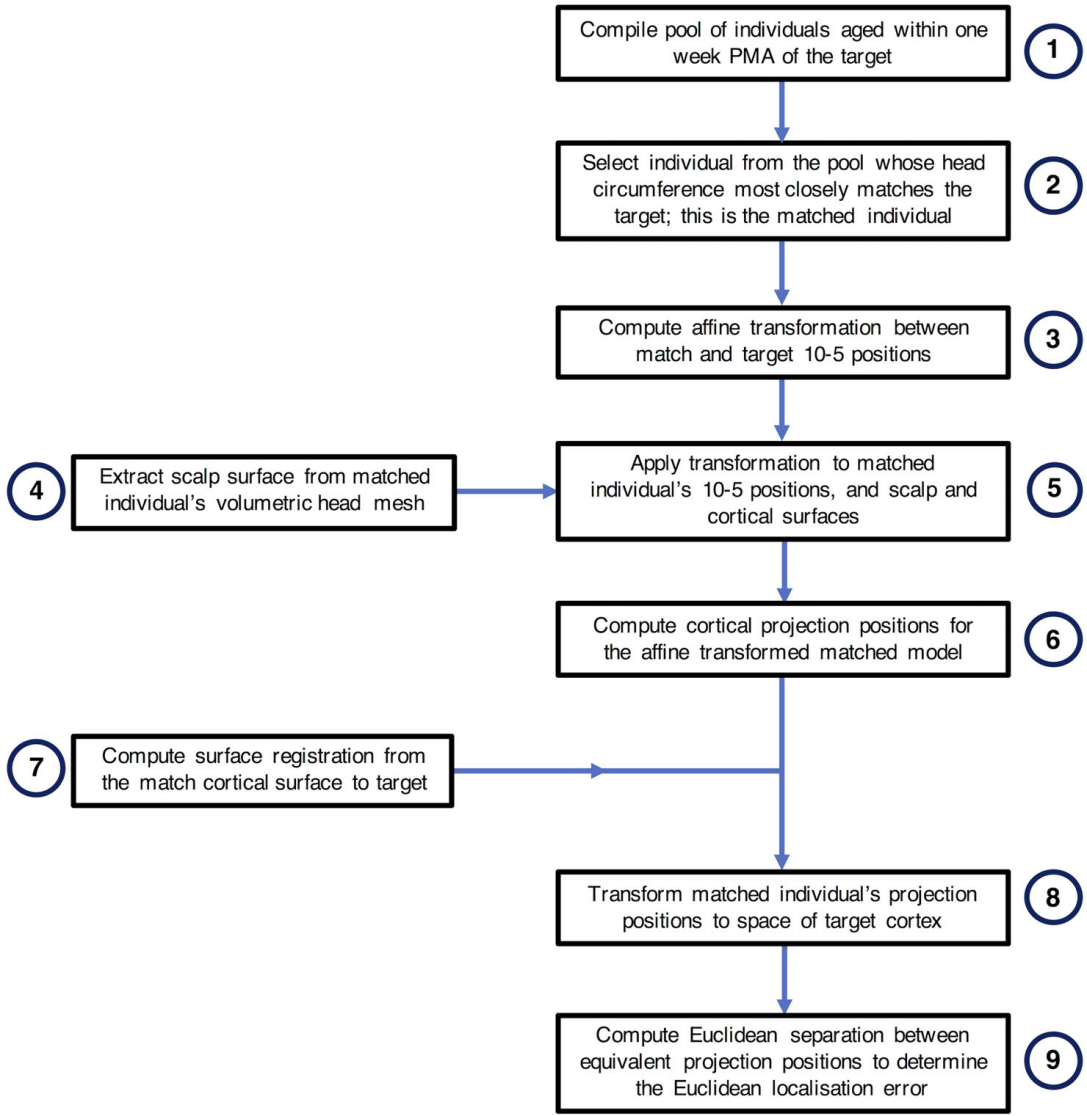
Flowchart of the process to compute the Euclidean localisation error for each individual acting as the target in a leave‐one‐out paradigm

Each individual in the database was selected in turn to act as the target in a leave‐one‐out paradigm—the process is shown in flow diagram for in Figure 3. A pool of infants with an age equal to that of the target subject, plus or minus 1 week, was compiled. The infant from this pool with the nearest head circumference to that of the target individual was chosen to be the target's match. An affine transformation matrix was defined between the 10–5 positions of the match and the target. This transformation was then applied to the matched model's 10–5 positions and the nodes of the volumetric and cortical surface meshes to register them to the space of the target. In a process identical to that described previously, the 10–5 positions of this registered, matched model were projected down to the cortical surface.

Using Multimodal Surface Matching, a spherical registration method that allows flexible alignment of a wide variety of different types of features on the cortical surface (Robinson et al., 2014; Robinson et al., 2018), the registration between the cortical surfaces of the target and the match were computed. Mean curvature features were chosen to drive the registration, as these features reflect the finer‐scale patterns of cortical folding (Bozek et al., 2018). In this work, surface registrations were computed between equivalent hemispheres (right and left) of the two different cortices.

For each projection of the matched individual, the cortical surface node nearest to the projection position was assigned a value of 1, while every other node of the cortical surface of the relevant hemisphere was assigned a value of 0. Based on the registration between the target and the match's cortical hemisphere, the values assigned to each node were resampled to the space of the target cortical surface mesh. The subsequent resampled node values then consisted of values between 0 and 1. To determine the transformed position of a given matched model projection on the cortical surface of the target, a weighted average was computed using the resampled value at each node as the weight for the position of each node.

This whole process results in two paired distributions of points in the coordinate space of the target cortical surface. These points represent the cortical locations underlying the 10–5 positions in the target individual (i.e., the “true” positions) and the equivalent positions one would obtain if the match is used instead of the subject‐specific structural prior. The Euclidean distance between each target projected position and their matched equivalent in the space of the target brain is defined as the Euclidean localisation error.

For each individual aged 41 weeks PMA (*n* = 43), the localisation error values associated with each projection position were interpolated to produce a map of localisation error as a function of position on the cortical surface. These individual localisation error cortical maps were transformed and resampled to the space of an age‐appropriate cortical surface atlas (constructed by Bozek et al. (2018)) using Multimodal Surface Matching, and the mean and *SD* of the node‐wise localisation error values were computed.

#### 2.2.1 Scalp‐to‐brain distance and extra‐cerebral tissue thickness

The vectors originating at the scalp surface used for computing cortical projections were also used to compute the scalp‐to‐brain distance, extra‐cerebral thickness and CSF thickness underlying each 10–5 position. Initially, it was noticed that the distribution of scalp to brain distances for a given individual rarely exceeded 30 mm; however, a small population of scalp locations lateral to the sagittal reference curve at the anteroinferior‐most regions of the head appeared at 40 mm and beyond. In such cases, the projection from the scalp surface did not intersect with the cortical surface that was immediately underlying the scalp position. As such, if the ray intersected with the cortical surface at a distance greater than 35 mm, this was deemed to be misleading, and such projection positions were assigned the position of the nearest cortical surface node to the scalp location.

The thickness of the extra‐cerebral tissue was computed as the distance to intersection between each ray vector from the 10–5 positions to the outer CSF surface extracted from the volumetric mesh. In cases where the nearest node method was used for the cortical projection, extra‐cerebral tissue thickness was calculated as the distance to the nearest CSF node. The CSF thickness underlying each 10–5 position was computed as the scalp‐to‐brain distance minus the extra‐cerebral tissue thickness.

## 3 RESULTS

### 3.1 Model characteristics

#### 3.1.1 Scalp segmentation

**FIGURE 4:**
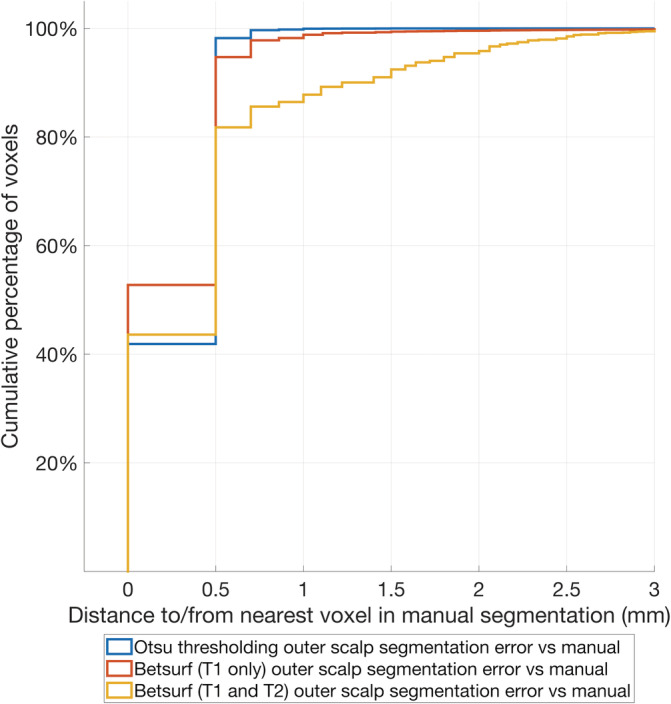
Cumulative distribution plot of the distances to/from the centre of each manually segmented boundary voxel from/to the centre of the nearest automated segmentation boundary voxel for each of the three automated methods. These distances are used as a measure of the error of the outer scalp boundary relative to manual segmentation

Three different methods to determine the outer scalp boundary were compared to manual segmentations for a subset of 12 individuals from the database, consisting of three different infants at 32, 36, 40, and 44 weeks PMA. Compared to manual segmentation, the mean surface distance across all individuals of all ages was lowest among those segmented with Betsurf using the T1‐weighted image only (0.266 mm, standard deviation (*SD)* 0.367 mm), closely followed by Otsu thresholding, whose *SD* was lower (0.295 mm, *SD* 0.254 mm), followed by Betsurf using both T1‐ and T2‐weighted images (0.466 mm, *SD* 0.631 mm). The Hausdorff distance (4.30, 12.5, and 13 mm for Otsu thresholding, Betsurf with T1 only, and Betsurf with both T1 and T2, respectively) and the modified Hausdorff distance (0.5, 0.707, and 1.87 mm for Otsu thresholding, Betsurf with T1 only, and Betsurf with T1 and T2, respectively) are lower for Otsu thresholding, in addition to its lower surface distance *SD*. Cumulative distribution functions displaying the data for all three automated segmentation methods compared to manual are shown in Figure 4.

**FIGURE 5:**
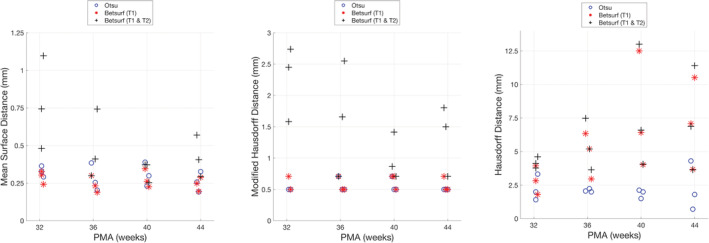
The metrics used to evaluate the automated segmentation methods are shown for each infant in the subset evaluated with manual segmentation

**FIGURE 6:**
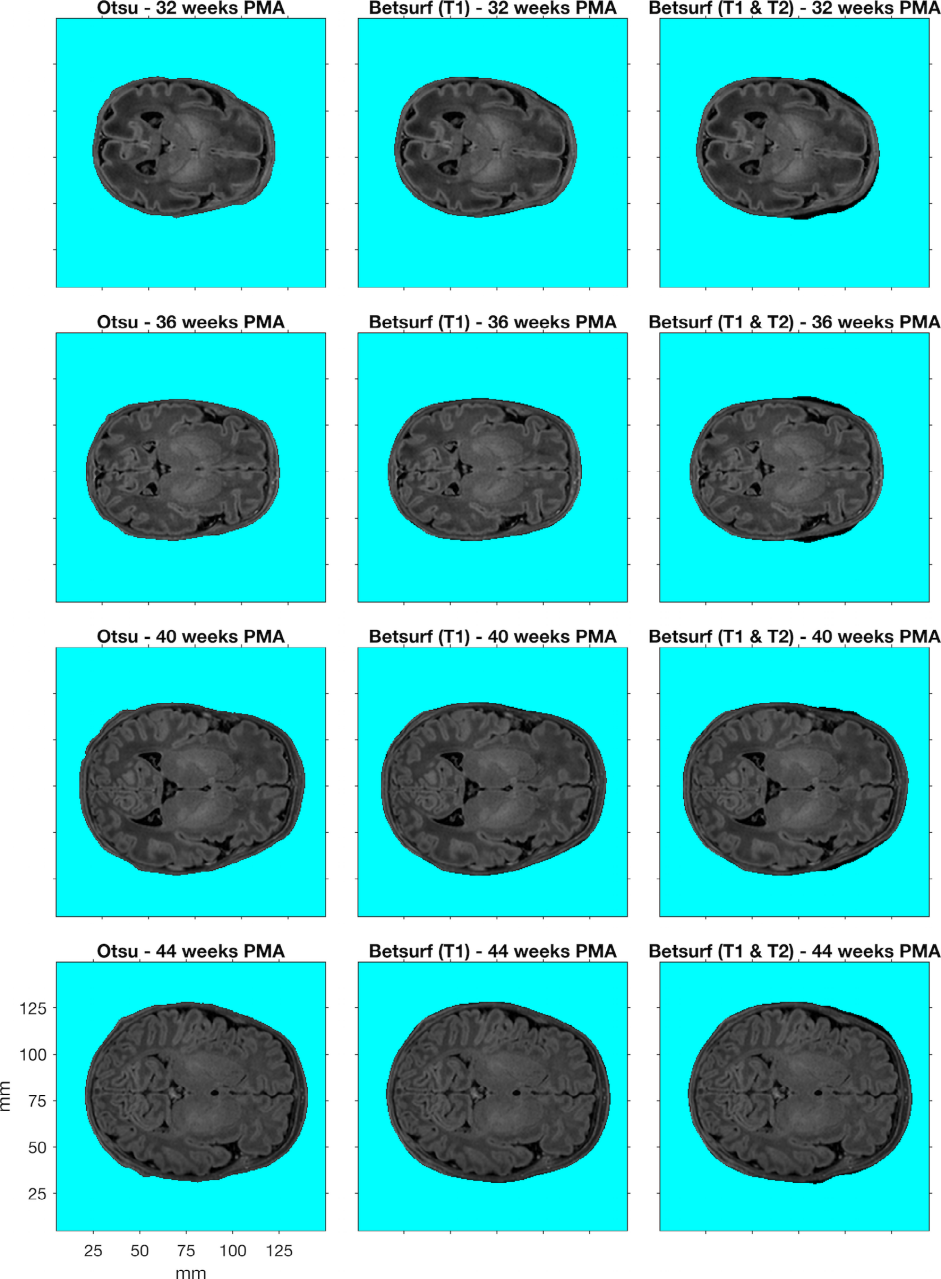
T1‐weighted images from example individuals at 32, 36, 40, and 44 weeks postmenstrual age (PMA). For each individual, the outer scalp boundaries determined using three different automated segmentation methods are shown (demarcated by the turquoise background)

The mean surface distance, modified Hausdorff distance and Hausdorff distance for each segmentation from each individual in the manually segmented subset is shown in Figure 5, and the resulting outer scalp boundaries from the different automated segmentation methods can be visualised in Figure 6. The modified Hausdorff distance indicates that Betsurf using both T1‐ and T2‐weighted images is less reliable than the other two methods at all ages, while the Hausdorff distance indicates Otsu thresholding to be the most consistent at each age investigated. A statistically significant but very slight negative correlation was found between Otsu segmentation error versus manual and age (*r* = −.085, *p* < .001). However, given its consistent performance and the fact that the mean surface distance was always less than 1 mm, we determined that it is appropriate to apply Otsu segmentation to structural data across the range of ages covered in the database.

#### 3.1.2 Database of individual structural priors

**FIGURE 7:**
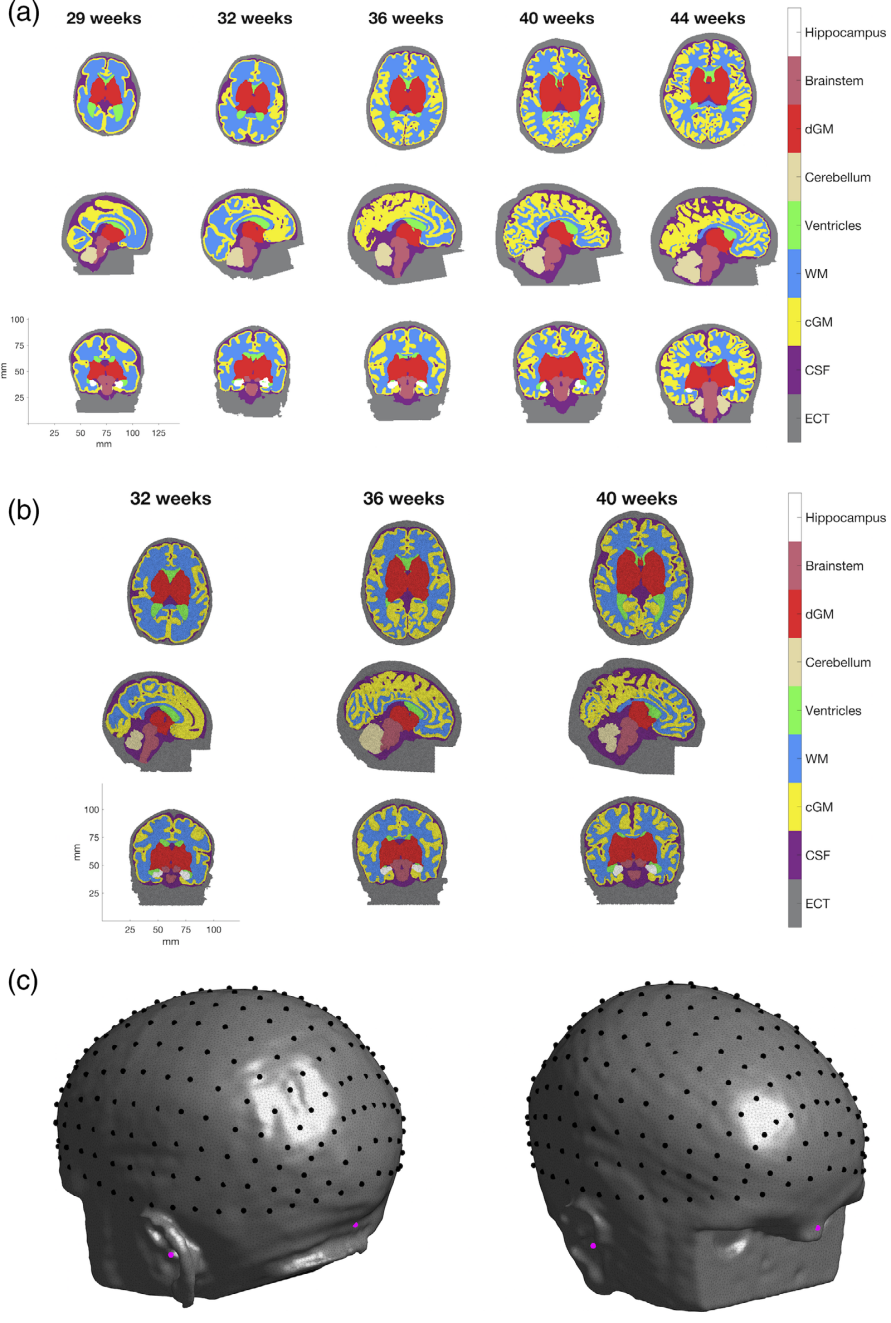
Example of multi‐layered (a) tissue masks and (b) meshes from neonatal infants aged 29–44 weeks postmenstrual age (PMA) (see colourbar). Tissues represented are extra‐cerebral tissue (ECT), cerebrospinal fluid (CSF), cortical grey matter (cGM), white matter (WM), ventricles, cerebellum, deep grey matter (dGM), brainstem, and hippocampus. The 10–5 positions on the scalp surface from an example infant aged 41 weeks PMA are shown in (c) in black, while the cranial landmarks are shown in magenta

Example components from the completed database of individual multi‐layered structural priors for neonates at different ages are shown in Figure 7. The cranial landmarks and 10–5 positions for an example individual are also shown in Figure 7. Table 1 summarises the properties and quality indices of the volumetric meshes. The number of nodes increases with age as one would expect given the increase in head volume, observable in Figure 7. Across all volumetric meshes in the database, 81.0% of elements exhibit a *q*_*vol*_ value of 0.7 or greater, indicating that the majority of elements are close to being equilateral. The mean Voronoi volume of the volumetric mesh is 0.474 mm^3^, indicating the high density of the mesh.

**TABLE 1:** Features of the volumetric meshes for each age in the database

Age	Mean q_vol ± *SD*	Mean Voronoi volume ± *SD* (mm^3^)	Mean *N* nodes ± *SD* (×10^6^)	Mean *N* elements ± *SD* (×10^6^)	Mean *N* faces ± *SD* (×10^6^)	*N* subjects
29	0.803 ± 0.115	0.473 ± 0.210	0.62 ± 0.00	3.59 ± 0.00	1.06 ± 0.00	1
30	0.802 ± 0.116	0.468 ± 0.210	0.63 ± 0.11	3.67 ± 0.66	1.08 ± 0.12	4
31	0.802 ± 0.116	0.466 ± 0.210	0.70 + 0.22	4.12 ± 1.32	1.22 ± 0.33	2
32	0.800 ± 0.117	0.472 ± 0.210	0.77 ± 0.04	4.50 ± 0.20	1.35 ± 0.78	3
33	0.802 ± 0.116	0.474 ± 0.210	0.82 ± 0.10	4.81 ± 0.62	1.40 ± 0.13	10
34	0.802 ± 0.116	0.476 ± 0.208	0.95 + 0.07	5.55 ± 0.40	1.60 ± 0.10	10
35	0.802 ± 0.116	0.477 ± 0.209	1.05 ± 0.08	6.16 ± 0.49	1.78 ± 0.11	16
36	0.801 ± 0.116	0.476 ± 0.208	1.07 ± 0.14	6.29 ± 0.83	1.83 ± 0.18	16
37	0.801 ± 0.117	0.475 ± 0.207	1.17 ± 0.08	6.90 ± 0.50	2.01 ± 0.12	13
38	0.799 ± 0.117	0.482 ± 0.206	1.20 ± 0.11	7.10 ± 0.67	2.12 ± 0.16	17
39	0.800 ± 0.117	0.477 ± 0.207	1.34 ± 0.11	7.94 ± 0.67	2.31 ± 0.17	19
40	0.800 ± 0.117	0.479 ± 0.206	1.36 ± 0.09	8.06 ± 0.52	2.37 ± 0.10	26
41	0.799 ± 0.117	0.479 ± 0.206	1.39 ± 0.10	8.21 ± 0.58	2.44 ± 0.14	39
42	0.799 ± 0.117	0.468 ± 0.205	1.46 ± 0.13	8.69 ± 0.75	2.58 ± 0.21	24
43	0.799 ± 0.117	0.472 ± 0.206	1.54 ± 0.12	9.15 ± 0.73	2.73 ± 0.22	8
44	0.798 ± 0.117	0.471 ± 0.204	1.56 ± 0.12	9.27 ± 0.73	2.78 ± 0.24	7

#### 3.1.3 Anatomical features

**FIGURE 8:**
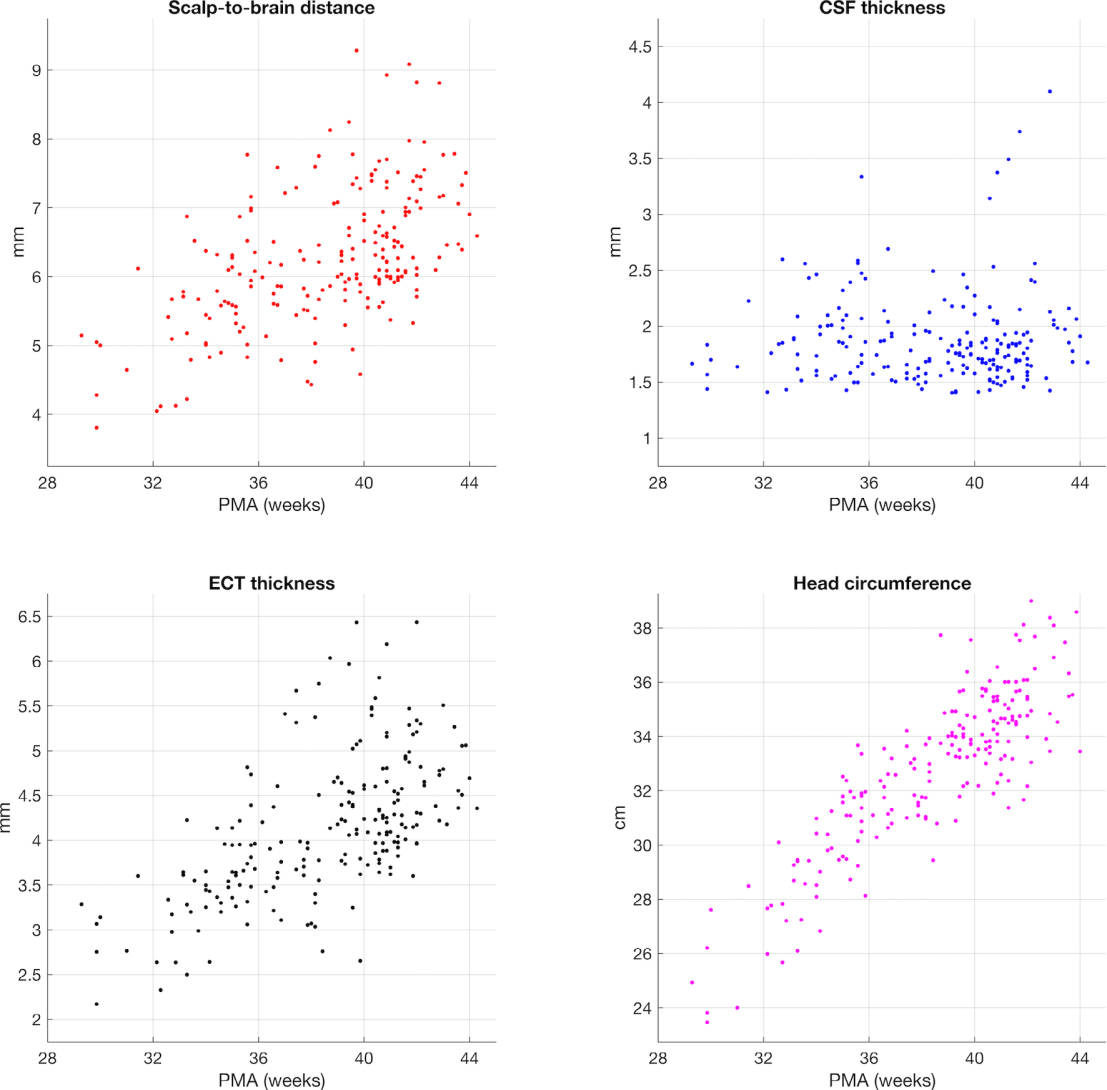
Median tissue thickness values underlying the 10–5 positions and head circumference as a function of age for all individuals in the database

**FIGURE 9:**
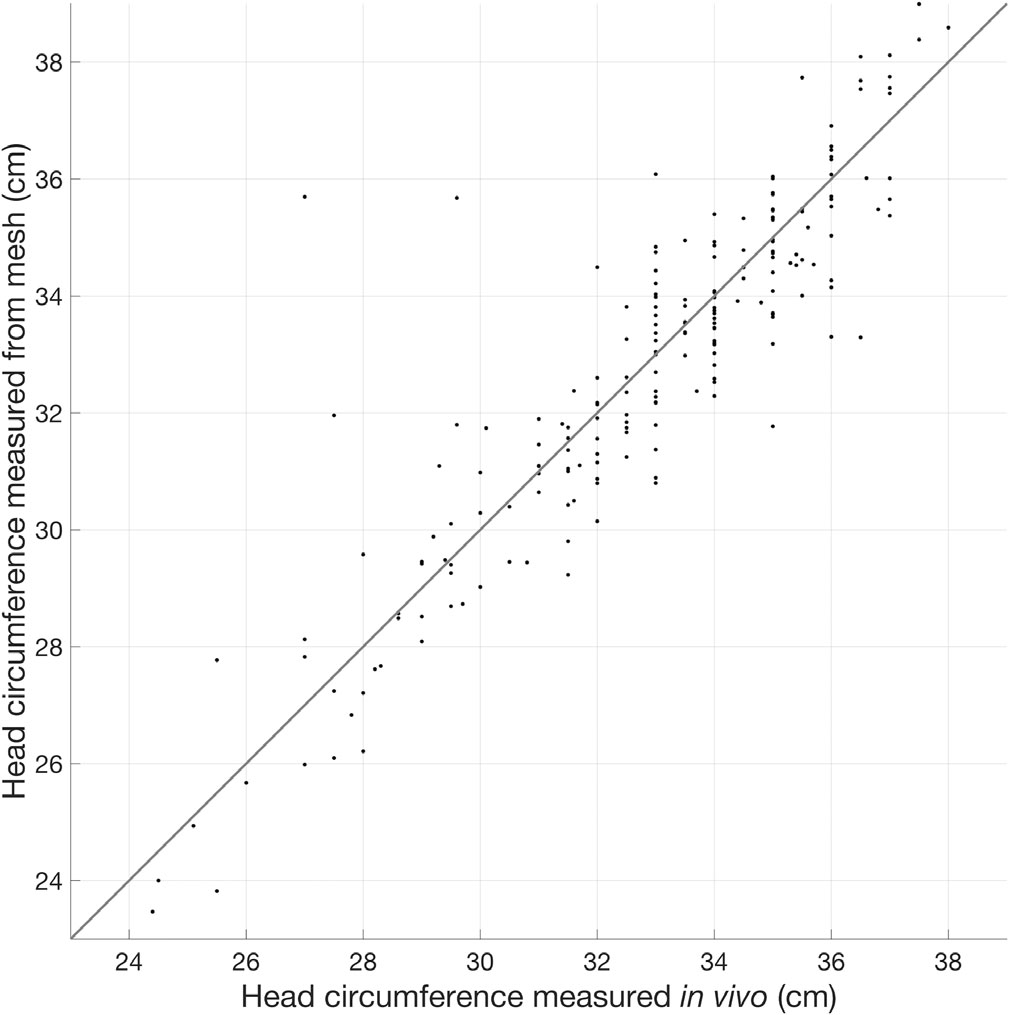
Head circumference measurements taken in vivo (x‐axis) plotted against head circumference measurements taken from volumetric meshes (y‐axis). The line of one‐to‐one proportion is shown

Figure 8 shows the median thickness of the extra‐cerebral tissue, the thickness of the CSF, and the median scalp‐to‐brain distance underlying the 10–5 positions for each individual as a function of age. Mesh‐derived head circumference as a function of age is also shown in Figure 8. For 204 of the 215 infants in the database, data were available for the head circumference as measured at time of scan. Figure 9 displays the measured head circumference plotted against the head circumference derived from the mesh for these individuals. There is a high correlation between the two sets of measurements (*r* = .90, *p* < .001).

### 3.2 Performance of the model in an example application

**FIGURE 10:**
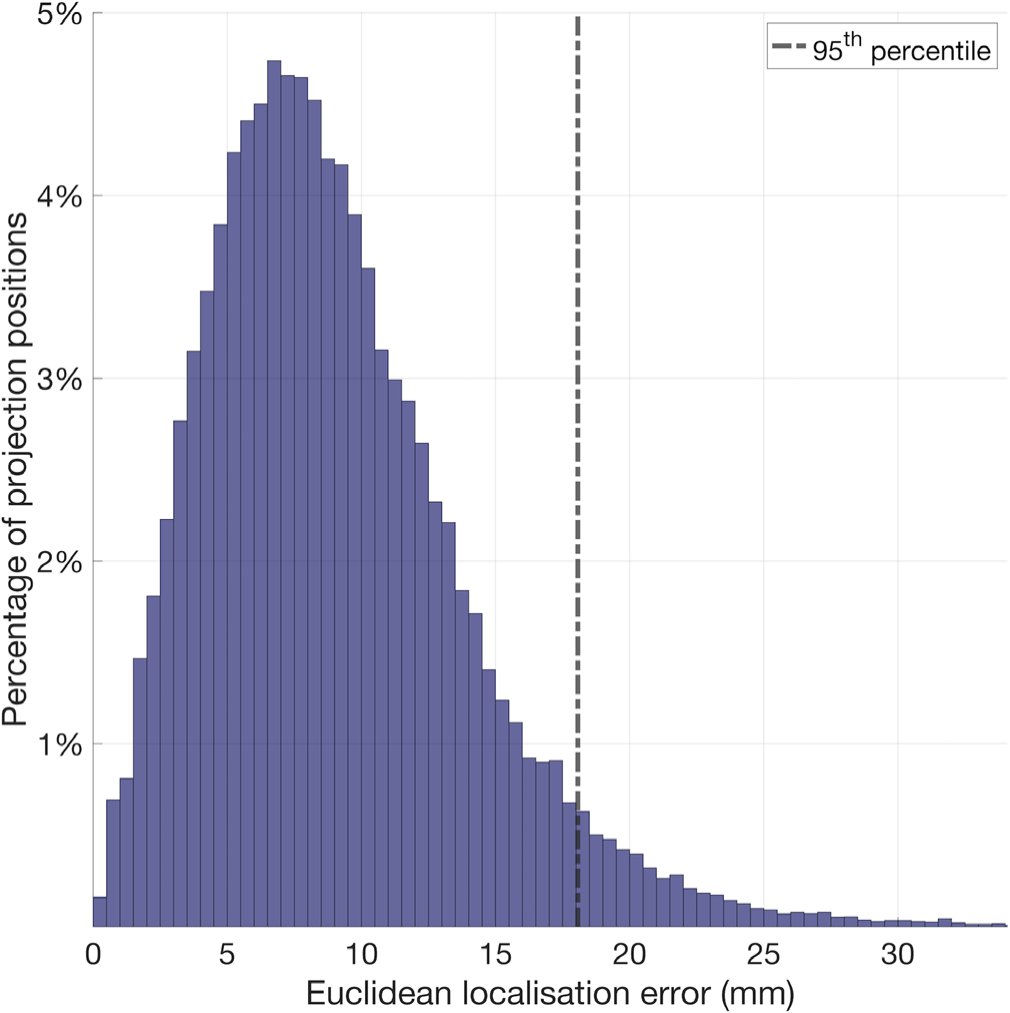
Histogram displaying the distribution of the Euclidean localisation error of each cortical projection position, combining data from each individual

**FIGURE 11:**
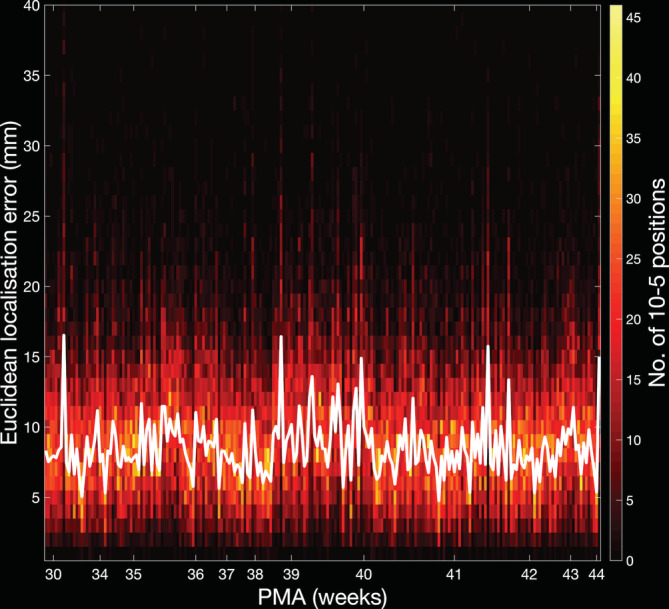
Histogram displaying the distribution of the Euclidean localisation error of the cortical projection positions per individual (see colourbar) and the median values for each individual (plotted as a white line)

The mean Euclidean localisation error across all projections from the 10–5 positions was determined to be 8.3 mm (median absolute deviation 3.8 mm), while 95% of projection points are within a Euclidean localisation error of 18.1 mm or less (see Figures 10 and 11).

**FIGURE 12:**
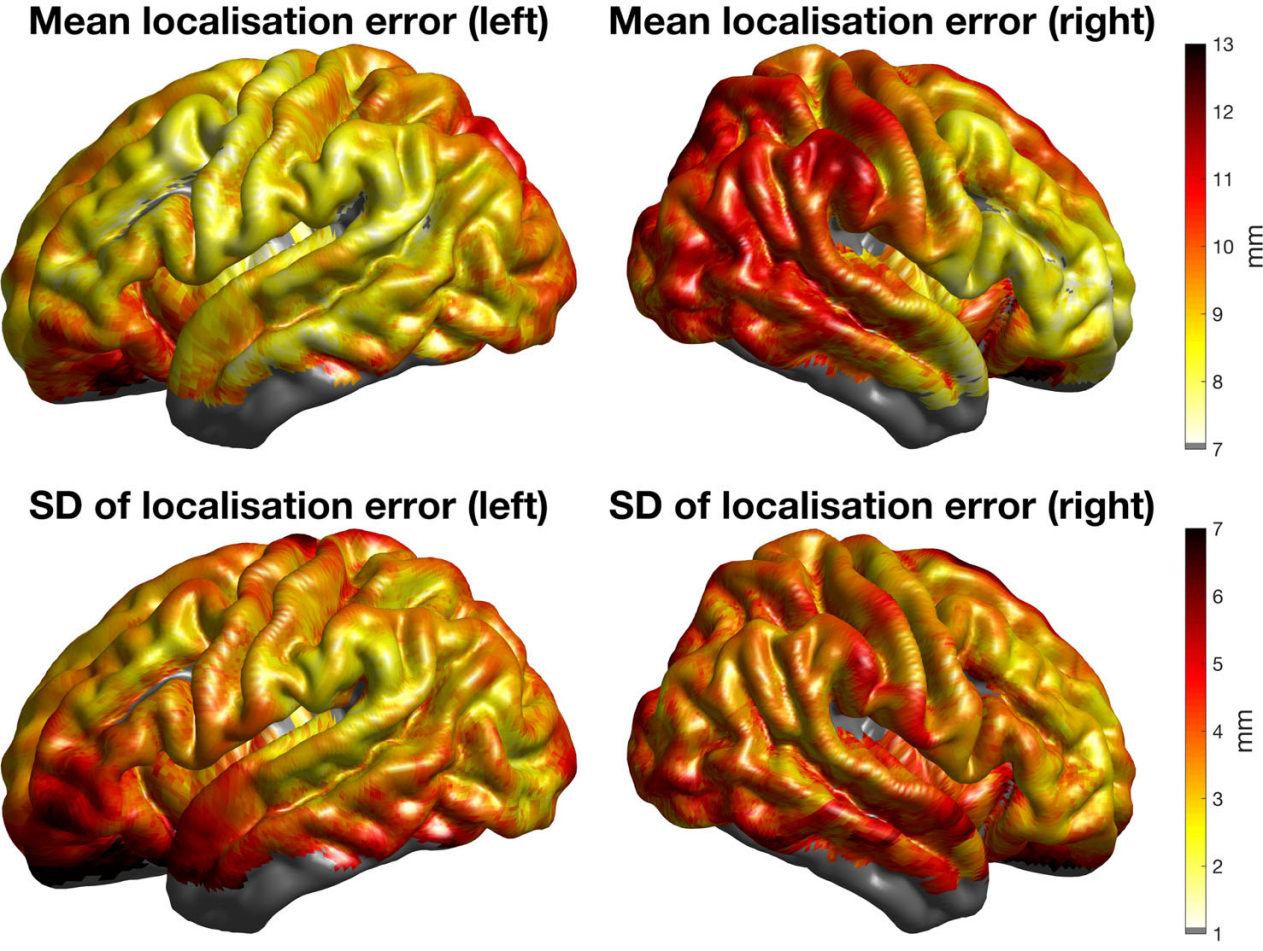
For each individual aged 41 weeks postmenstrual age (PMA), the Euclidean localisation error was interpolated across the cortical surface using the value at each projection position. The interpolated localisation error maps for each of these individuals were registered to the surface of a 41‐week PMA cortical surface atlas and averaged. Here, the node‐wise values of the mean and *SD* are displayed

The localisation error cortical map at 41 weeks PMA is shown in Figure 12. The localisation error is lowest anteromedially (except for cortical areas proximate to the longitudinal fissure) and is highest posteromedially. A localisation error of 11 mm or below is apparent across almost the entirety of the sensorimotor cortex (except proximate to the longitudinal fissure) and over the vast majority of the superior and middle temporal gyri bilaterally. The node‐wise *SD* of the localisation error is 4.5 mm or below for the vast majority of the sensorimotor cortex and the majority of the cortical surface, while the overwhelming majority of the temporal lobe has a *SD* below 5.5 mm.

## 4 DISCUSSION

In this work, we have described the production of a database of 215 individual structural priors using data from infants aged 29–44 weeks PMA for use in a range of neuroimaging modalities. For each individual, our data package includes:

- A voxelised mask of tissues of the head volume.
- A tetrahedral mesh of tissues of the head volume.
- A scalp surface mesh.
- The coordinates of the 10–5 positions and cranial landmarks.
- Cortical surface and white matter meshes, taken from the dHCP processing pipeline and re‐aligned to the space of the tetrahedral meshes.

More datasets will be made publicly available by the dHCP in future, and so we plan to subsequently add more head models to our database once the dHCP datasets are released publicly.

Ideally, a structural prior would be derived from an individual's own MRI scan; however, this is often not available and requiring each individual to undergo an MRI scan undermines the benefits of many functional imaging techniques that enable neonates to be studied at the cot‐side. The use of an atlas removes the need for each subject to undergo an MRI scan, addressing the fundamental challenge posed by imaging modalities that do not simultaneously acquire structural and functional data.

The structure of the neonatal brain is highly variable across ages and individuals, which is evident from Figure 7 (see Section 3), displaying example structural models at different ages, with substantial structural changes occurring from 29 to 44 weeks PMA. A study of over 300 infants aged 28–44 weeks PMA (Makropoulos et al., 2016) found that the relative volume of white matter decreases during this period while the relative volume of cortical grey matter increases. (Makropoulos et al., 2016) also found that cortical surface area increases with age. The use of a single model for infants across this age range is therefore inappropriate. As stated in Section 1, previous work has detailed the construction of age‐specific atlases that address these structural changes. However, little is known about age‐matching population‐level atlases of the head, or the relation between age‐ and size‐matching.

Spatially averaging structural data to produce population‐level atlases of the neonatal head results in diminished detail of gyrification, and so such models of the head do not offer an anatomical volume representative of an individual head in which a field, such as the spatial distribution of photon migration, can be modelled. The database of structural priors of individual‐level anatomy produced in this work permits the development of a targeted atlas for a given subject that is likely to be more representative than a spatially averaged model exhibiting diminished gyrification. For a given subject, an individual from the database can be matched on a granular level, based on (for instance) head size, head shape, age, gender, and the relative positions of landmarks on the scalp. The fact that individual‐level data is being used means that gyri and sulci remain present in the structural priors, which is truly representative of individual anatomy.

The structural priors presented in this work can be used in many functional neonatal imaging modalities. These could be used as a space to which functional data can be registered, or to solve the forward problem in, for example, EEG, MEG, NIRS, and DOT (Arridge & Cooper, 2015; Azizollahi et al., 2016; Legon et al., 2018; Mueller et al., 2017; Pirondini et al., 2018; Ranjbaran et al., 2019; Roche‐Labarbe et al., 2008; Routier et al., 2017). To do this, it is necessary to determine the position of sources and sensors placed on the scalp surface of a given subject, such as optodes or electrodes. These can be computed in relation to or in correspondence with the 10–5 positions provided in our data package, or through the use of a magnetic positioning device or photogrammetry methods. In addition, the cranial landmarks are provided for each model in the database; these landmarks can also be obtained from a subject at the cot‐side or bed‐side using an electromagnetic positioning device or photogrammetry methods and can be used to register a structural prior to the dimensions of a subject.

In the case of DOT, a forward model of photon transport is computed by modelling photon propagation from sources to detectors through the volume provided by the structural prior, producing a model of how the measured optical intensity will change given a change in the optical properties in the head. The inversion of this forward model provides an estimate of how optical properties in the head change given a change in detected intensity, which can then be combined with optical data to yield an image of those changes in optical properties (Arridge & Cooper, 2015).

Appropriate properties need to be assigned to the nodes (or elements) of the structural prior, which may vary in correspondence with tissue type as is the case for optical properties, magnetic permeability and electrical conductivity. For the volumetric structural priors (tissue masks and tetrahedral meshes) presented in this work, we provide tissue assignments for the extra‐cerebral tissue in addition to the eight intra‐cerebral tissue types of grey matter, white matter, and CSF as defined through the dHCP structural pipeline (Makropoulos, Robinson, et al., 2018). Though the motivations for this work arise from our research in DOT, the constraints imposed by a lack of structural information is not unique to DOT, and it is hoped that the structural priors presented in this work will prove useful in other imaging fields.

### 4.1 Extra‐cerebral tissue segmentation

In this work, we have endeavoured to ensure that the head models produced represent the realistic anatomy of individual infants and quantify the error associated with extra‐cerebral tissue segmentation. In order to build the database, a series of methods for automated segmentation of the extra‐cerebral tissue were evaluated by comparison with manual segmentations from a subset of individuals. The error associated with the use of our preferred method (Otsu thresholding) is typically less than 0.5 mm at the outer scalp boundary, which is acceptable given the resolution of the associated MRI images and the typical resolution of DOT (Cooper et al., 2012; Ferradal et al., 2014, 2016).

Few publications exist that focus on the segmentation of neonatal extra‐cerebral tissue. Ghadimi et al. (2008) produced probabilistic atlases for scalp and skull using T1‐weighted images from three subjects aged 39–42 weeks PMA that were manually segmented and transformed to the space of the GRAMFC atlas (Kazemi, Moghaddam, Grebe, Gondry‐Jouet, & Wallois, 2007). To segment scalp and skull for a given subject, the T1‐weighted image was normalised to the GRAMFC atlas, and the probabilistic scalp and skull atlases were then used to identify scalp and skull points, followed by a level‐set based reconstruction to obtain closed surfaces. Daliri et al. (2010) applied a similar method by constructing a probabilistic atlas whereby six images (normalised to the space of the GRAMFC atlas) were manually segmented to produce atlases for scalp and skull tissues. A Bayesian classifier was then used to weight local features of the MR image against those of the probabilistic atlas, with the weighted features then being fed to a Hopfield Neural Net to obtain an estimation of the skull layer.

The database of neonatal head models we present was made using previously validated segmentations of neonatal MR images that retained brain tissues and CSF (Makropoulos, Robinson, et al., 2018). As such, the outer boundary of the cerebral tissues was taken as the inner skull boundary, and so only the outer scalp boundary was sought to produce a combined skull and scalp segmentation; the extra‐cerebral tissue. The studies described above present a method to separate skull and scalp tissue in infants, however the methods would be unfeasible for the purposes of our work. First, the authors rely on manual segmentation of the outer and inner skull boundaries, a time‐consuming method which can only provide data from a small number of individuals in a reasonable time frame. Second, the skull/scalp boundary is difficult to discern on a neonatal MRI scan, rendering such methods both subjective and difficult to validate. Further, for optical applications, the optical properties of the skull and scalp are relatively similar (absorption coefficient and reduced scattering coefficients of 0.018 and 1.9 mm^−1^ for scalp, and 0.016 and 1.6 mm^−1^ for skull (Dehaes et al., 2013)).

Despite this, the lack of distinct segmentations for skull and scalp in our models remains a limitation of this work, particularly for EEG applications where scalp and skull have different conductivity properties (Azizollahi et al., 2016; Roche‐Labarbe et al., 2008; Routier et al., 2017). Another limitation to note with regard to the extra‐cerebral tissue is that our models do not include any information on the structure of the fontanels. With regard to optical properties, it is known that the inclusion of the fontanel in a head model improves the accuracy of the recovery of absorption changes (Dehaes et al., 2013) and is known to improve source localisation in EEG (Roche‐Labarbe et al., 2008). Computed tomography data is required to discern the fontanels from bone (which cannot be achieved with MRI data alone) and such data did not exist for the individuals whose data were used to produce the database presented in this work. Some literature values are available for the thickness of extra‐cerebral tissue and CSF, though they are somewhat difficult to compare to the current work. (Beauchamp et al., 2011) conducted a study of the brain‐to‐scalp distances of subjects aged from birth to 12 years, derived from MRI data. This included data from 14 neonates, which yielded a highly variable pattern of brain‐to‐scalp distances, with mean values ranging from approximately 5 to 10 mm between individuals. (Brigadoi & Cooper, 2015) computed the extra‐cerebral tissue and CSF thickness underlying each surface node as well as overall scalp‐to‐brain distances for each age of the Brigadoi et al. head model (Brigadoi et al., 2014). The median values of CSF thickness of the Brigadoi et al. model are notably higher than those for individuals from our database at corresponding ages. The opposite is true for extra‐cerebral tissue thickness, which is consistent with the author's assertion that their models likely underestimate the thickness of the extra‐cerebral tissue. The close correspondence demonstrated here between segmentations completed manually and by our preferred automated method, as well as the high correlation between measured and mesh‐derived head circumference measures, underpins our confidence that the models in our new database accurately represent extra‐cerebral tissues.

Over half of the prospective datasets (366 of 634) acquired as part of the dHCP cohort and used in this work had to be excluded. The reason for the vast majority of these exclusions (86% of 366) was that the extra‐cerebral tissue extended out of the field of view in the T1‐weighted MR images. This is often found in clinical MRI scans where the duration of the scan is limited. In order to produce an accurate representation of non‐brain tissues, we required that all extra‐cerebral tissues were visible across the image volume. An interpolation method could potentially have been used to correct for the missing extra‐cerebral tissue in these excluded datasets. However, this process would have required its own optimisation and validation and, given the large number of individuals that were acceptable, it was determined that it was better to rely solely on complete structural datasets.

### 4.2 Error incurred by using an individual‐matched structural prior

This work describes the construction of a database of structural priors but it was also essential to quantify the error associated with an application of this database. For simplicity, we chose to test the utility of an individual‐matching approach using a leave‐one‐out analysis. At 41 weeks PMA, the localisation error associated with this application was approximately 11 mm or less across the majority of the motor cortex and the majority of the superior and middle temporal gyri bilaterally. The median localisation error across all 10–5 positions across all subjects is 8.3 mm. Assuming a circular areal distribution of error, this suggests a geodesic point spread function of approximately 216 mm^2^, with this extending to 460 mm^2^ with an increase of one median absolute deviation (3.8 mm) above the mean. For context, Bozek et al. report the average area of the posterior portion of the superior temporal gyrus to range from 1,175 mm^2^ at 36 weeks PMA to 1,525 mm^2^ at 44 weeks PMA. The frontal and temporal areas have been shown to be important in infant social development (Singh, Okamoto, Dan, Jurcak, & Dan, 2005; Tsuzuki et al., 2007), and our analysis provides evidence that using a matched brain from this database could potentially offer spatial precision at the gyral level in these areas.

There are three sources of error in atlas‐guided DOT, as stated by Cooper et al. (2012). The first is that there are anatomical differences between the matched model and the target subject. To reduce this error, future work will identify the factors that are the best indicators of a match, which may include (but are not limited to) head circumference, head size, age, gender, and features derived from the cranial landmarks such as nasion‐to‐inion distance. The second source of error is the imperfect registration of the matched model to the target's space. In this work, an affine registration was employed to spatially register the matched model to the target's space. In the adult, the error due to affine registration is estimated at 4–7 mm (Singh et al., 2005; Tsuzuki et al., 2007). It is difficult to disentangle the affine registration error from the error introduced due to anatomical differences and, as such, future work will need to investigate factors that could affect these sources of error in combination. In practice, it will be difficult to acquire all 10–5 positions. Feasibly, the registration of a structural prior to the subject space will rely on positions of cranial landmarks (Nz, Iz, Ar, Al, and Cz); as a result, a limitation of this work is the more constrained affine transformation as compared to real world applications of this process. The third source is the error inherently associated with image reconstruction in DOT. The example application described and tested in this work was deliberately simple so as to be generalisable across neuroimaging techniques, and so this work does not evaluate the error that image reconstruction could introduce.

A limitation of our approach is the determination of the positions of the cranial landmarks. There is ambiguity in the definitions of the cranial landmarks, leading to issues with reproducibility in determining their positions. This is particularly the case for the inion (Jurcak, Tsuzuki, & Dan, 2007). As such, there exists rater‐originated error in the determination of cranial landmarks positions and (by extension) the 10–5 positions calculated for each individual in the database.

### 4.3 Comparison to previous work

The database of structural priors presented in this work confers several advantages over the population‐level atlas produced by Brigadoi et al., the most realistic model available for use in place of a subject‐specific model. The database of structural priors presented in this work was completed using MRI data of a superior resolution (0.5 mm × 0.5 mm × 0.5 mm in this work, 0.86 mm × 0.86 mm × 1 mm in Brigadoi et al.), and the segmentation used to build the model were obtained using a more up‐to‐date segmentation algorithm (Makropoulos et al., 2014; Makropoulos, Robinson, et al., 2018). We have also included a validation of a method to segment the extra‐cerebral tissue which was not included in the work published by Brigadoi et al.

In addition, the models in the database are compatible with surface registration techniques. In this work, we have not included a comparison of the error incurred by using a matched individual atlas with the error incurred by using a population‐level neonatal atlas (such as that constructed by Brigadoi et al.). Given that the cortical surfaces in these models are smooth, there is very little local variation in sulcal depth and curvature. Therefore, one cannot rely on these features to yield a meaningful mapping from an individual cortical surface exhibiting gyrification to that of a population‐level atlas exhibiting gyrification to a much lesser degree. Such a registration would be prone to a high level of error which would be impossible to discern from the error incurred by using a population‐level atlas. This lack of gyrification means that the use of surface registration techniques such as FreeSurfer and Multimodal Surface Matching may not be appropriate to quantify the error incurred by using a population‐level atlas with respect to the use of a subject‐specific model. The compatibility of our models with these surface registration methods is a distinct advantage of the database of structural priors presented in this work as surface registrations can be used to meaningfully validate their application.

There exist cortical atlases, such as those constructed by Bozek et al. (used as a common space for analysis in this work), which make use of structural data at the population level and manage to preserve gyrification detail by averaging in the cortical surface space. However, in order to model the spatial distribution of a field within the head, a structural prior that represents a volume is required.

Macroanatomical labelling is of great benefit to functional neuroimaging methods as it allows an anatomical label to be associated with the location of cortical activation. The dHCP datasets used in this study include cortical parcellation maps that label 17 discrete cortical regions per hemisphere for each individual (Gousias et al., 2012; Makropoulos, Robinson, et al., 2018). These parcellation maps are also provided for each individual in our publicly available database. In addition, other neonatal cortical surface parcellation atlases, such as that published by de Macedo Rodrigues et al. (2015) and Alexander et al. (2017), can be registered to each individual using surface registration, expanding the range of parcellations that can be used to label the anatomical location of functional activation. Such parcellation atlases will be instrumental in order to apply our models to the study of, for example, functional connectivity.

### 4.4 Future work

In this study, we have described the construction and validation of a database of neonatal head models and have demonstrated a simple example application of how the resulting head model database can be applied. We have not sought to demonstrate best practice in using this database; in future we hope to test multiple different methods to determine what the best practice approach is for using an individual‐level head model database with DOT data. The database of structural priors presented in this work provides a novel opportunity for such ideas to be tested and validated.

We have not attempted to provide macroanatomical labelling of the positions of the cortical projections. Using a 12‐month‐old infant template, Tsuzuki et al. (2017) demonstrated that the 10–10 system (a lower density derivative of the 10–5 system) is sufficient to predict underlying macroanatomical cortical structures. Future work involving the database of structural priors described here could investigate whether such a consistent relationship is present in the neonatal population, using cortical projection and scalp projection protocols used in Tsuzuki et al. (2017), Kabdebon et al. (2014), and Matsui et al. (2014). This would aid the interpretation of the location of activation measured transcranially by modalities such as fNIRS and EEG.

The obvious limitation of implementing the database in this work through a matched‐individual approach is that the use of an individual atlas may bias the resulting images, since the structural prior does not incorporate anatomical variation across infants at a particular age. To address this bias, the database presented could also permit a probabilistic approach that makes use of head models from multiple individuals. For a given subject, a pool of closely matched individuals from the database could be compiled, and each of these head models could be spatially registered and used to reconstruct an image. The resulting reconstructed images could then be averaged in an arbitrarily chosen individual space using surface‐based registration techniques such as Multimodal Surface Matching and FreeSurfer, so that the resulting image is influenced by a degree of anatomical variation in a well‐targeted population without being biased towards a specific individual's anatomy. Alternatively, the pool of best‐matching individuals compiled from the database permits the production of a population‐level atlas from a more demographically constrained cohort.

## 5 CONCLUSION

We have described the construction of a database of multi‐layered, individual‐level models of the neonatal head for infants aged 29–44 weeks PMA, and have demonstrated a simple application of this database. Given that no similar database exists for neonatal head anatomy at the individual level, we anticipate that this database will be of use across a range of neuroimaging, neuromonitoring, and neurostimulation techniques, and particularly in DOT. In future, we aim to evaluate different applications of the database to determine best practice to find a matching head model or a subset of matching head models that minimises the localisation error. This database is now freely available at www.ucl.ac.uk/dot-hub.

## CONFLICT OF INTEREST

The authors declare no conflicts of interest.

## Data Availability

The data that support the findings of this study are available as part of the data release of the Developing Human Connectome Project at http://www.developingconnectome.org/project/datarelease‐user‐guide/ and http://www.developingconnectome.org/second-data-release/, the final reference in the references list in the manuscript and is available in the public domain. The database of head models constructed as part of this work which support the findings will be available at www.ucl.ac.uk/dot-hub following an embargo from the date of publication.
